# How work and family dynamics shape mental health across the life course: Insights from a longitudinal study in Japan

**DOI:** 10.1016/j.ssmph.2026.101920

**Published:** 2026-04-03

**Authors:** Nobutada Yokouchi, Hiroshi Ishida

**Affiliations:** a Graduate School of Human Development and Environment and Advanced Research Center for Well-being, Kobe University, Kobe, Japan; b Institute of Social Science and Graduate School of Agricultural and Life Sciences, University of Tokyo, Tokyo, Japan

## Abstract

• Family status changes modified the within-person impact of the psychosocial work environment on women's mental health.
• Entering into marriage strengthened the positive association between flexible work arrangements and women's mental health.
• Acquiring a parental role weakened the positive association between flexible work arrangements and women's mental health.
• The mental health impact of the psychosocial work environment remained stable for men, regardless of family status changes.

## 1 Introduction

A substantial body of research has demonstrated that both work and family domains affect the psychological well-being of the working population (Greenhaus and ten Brummelhuis, 2013; Kinnunen et al., 2024). In the work domain, the Job Demands–Resources (JD–R) model is a widely used framework that explains how the psychosocial aspect of the work environment influences workers’ well-being (Demerouti et al., 2001; Bakker & Demerouti, 2017). Specifically, the JD–R model posits that job characteristics can be categorized as either job demands or resources. Job demands are “the physical, psychological, social, or organizational aspects of the job that require sustained physical, cognitive, and/or emotional effort and are therefore associated with certain physiological and/or psychological costs” (Bakker & Demerouti, 2007, p. 312). Examples of job demands include workload and time pressure, which are linked to negative mental health consequences such as burnout (Bakker et al., 2023). Job resources are “the physical, psychological, social, or organizational aspects of the job that have motivating potential, that is functional in achieving work goals, that regulate the impact of job demands, and that stimulate learning and personal growth” (Bakker & Demerouti, 2007, p. 312). Examples of job resources include social support and job control, which are associated with positive mental health consequences such as work engagement (Bakker et al., 2023).

In the family domain, the association between family status and mental health has been widely investigated among the general population. Prior research suggests a positive effect of entering into marriage because married individuals often benefit from socioemotional and economic resources that contribute to better health outcomes (Umberson et al., 2013; Umberson & Thomeer, 2020). Transition out of marriage, such as marital dissolution, is generally detrimental to health due to diminished resources and the inherently stressful nature of the event. The effect of transition to parenthood on mental health is contingent on demographic, socioeconomic, and cultural contexts. Parenthood can be either demanding or rewarding, depending on various factors such as gender, marital status, and adherence to intensive parenting ideology (Umberson et al., 2013; Nomaguchi & Milkie, 2020).

In addition to these separate bodies of literature, the interrelationship between work and family domains has received increasing scholarly attention amid substantial global changes over the last few decades. The rising female labor force participation and the corresponding increase in dual-earner couples have made it more challenging for many workers to balance their work and family responsibilities (Ruppanner, 2015). The shift toward a knowledge- and service-based economy and the development of information and communication technologies have increased autonomy and flexibility regarding when, where, and how work is performed, thereby blurring the boundaries between work and family domains (Major & Germano, 2006). Consequently, a growing body of research has focused on the perception of work–family interaction in terms of its valence (positive or negative), degree (strong or weak), and direction (work-to-family and/or family-to-work) (Kinnunen et al., 2024). Empirical evidence continues to accumulate regarding the association between work–family interaction and mental health; for example, negative work-to-family spillover, often referred to as work–family conflict, has been linked to adverse mental health outcomes such as burnout and psychological distress (Gisler et al., 2018).

Some studies have further elaborated on the specific circumstances within the work and family domains that shape experiences of work–family interaction, an approach referred to as the cross-domain approach (Greenhaus and ten Brummelhuis, 2013). For example, in a cross-sectional study of Korean employees, Kim et al. (2019) found that the association between quantitative workload and job control, on the one hand, and resulting distress, on the other, varied depending on marital and parental status. Longitudinal studies on this cross-domain approach are also emerging, often guided by the life course perspective (Elder et al., 2003; Moen & Sweet, 2004). The life course perspective provides a theoretical framework for examining the dynamic interplay between work and family trajectories over time (Han & Mortimer, 2023). For example, Sabbath et al. (2015) developed life course typologies combining job demands, job control, and family status among American working mothers. They found that women who became single mothers later in life and experienced low job control had the highest mortality rates. Despite these advancements, there remains a scarcity of research on how the psychosocial work environment and family status interact to shape the mental health of workers over the life course. Therefore, the present study examines whether family status modifies the association of the psychosocial work environment with mental health among men and women in Japan. The focus here is on family status rather than family events because our interest is in the sustained impact of occupying these roles as a spouse or a parent, and how the demands and resources that accompany these roles modify the relationship between the psychosocial work environment and mental health.

The primary theoretical framework guiding this study is the Work–Home Resources (W–HR) model (Ten Brummelhuis & Bakker, 2012). By assuming that people strive to acquire, retain, and protect their finite personal resources, such as time, energy, and attention (Hobfoll, 1989; Hobfoll et al., 2018), the W–HR model provides a unified framework linking the work and family domains by explaining how the dynamics between demands and resources in each domain shape individual well-being. The JD–R model can be placed within this overarching framework to depict the specific aspects of the work domain, i.e., job demands and job resources. These frameworks suggest a potential mechanism through which family status modifies the mental health impact of job demands and resources. First, when job demands are high, people have fewer resources available to meet demands in the family domain. If the family role introduces additional demands, such as caring responsibilities, these demands can further deplete personal resources and negatively impact mental health. In contrast, if the family domain provides resources, such as social support from spouses, these resources can help individuals manage job demands, thereby mitigating the depletion of personal resources and their impact on mental health. Second, job resources can help people accumulate their personal resources, supporting better mental health. If the family role provides additional resources, the accumulation of resources is further enhanced, thereby improving mental health. However, if family demands are present, job resources may be redirected to address those demands, potentially reducing their beneficial effect on mental health. Third, a critical perspective for our framework is the distinction between domain-specific and boundary-spanning resources within the work domain (Voydanoff, 2009), which are categorized together as job resources in the JD–R model. While resources such as job control and career-related support are typically confined to the work domain, resources such as flexible work arrangements facilitate the transfer of resources between work and home. Following the W–HR model, we expect that these boundary-spanning resources are more susceptible to family status than domain-specific resources when shaping mental health. Finally, we apply a life-course perspective as our analytical lens to examine dynamics between the work and home domains. Rather than viewing work and family as static states, this perspective emphasizes the interdependent trajectories of individuals over time. By separately focusing on between-person and within-person associations, we can more accurately capture how the mental health impact of the psychosocial work environment shifts as individuals navigate the major life transitions.

**Fig. 1:**
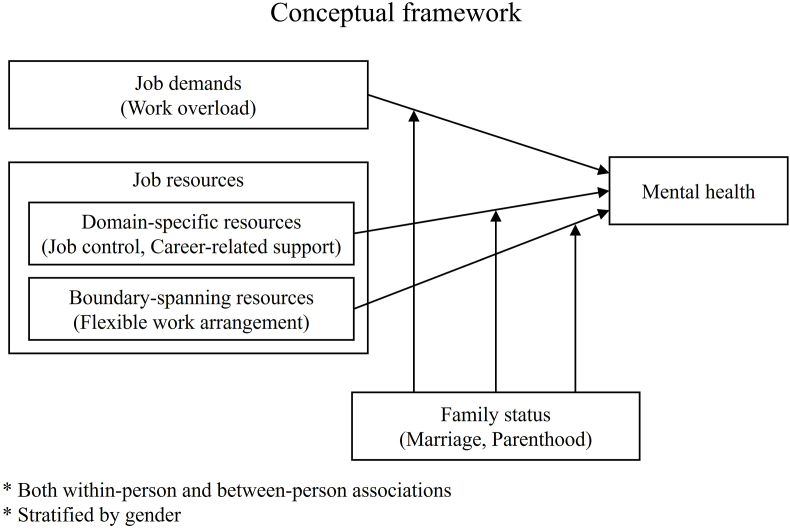
Conceptual framework.

The conceptual framework corresponding to our theoretical framework is summarized in Fig. 1. We conceptualize the psychosocial work environment, i.e., job demands (work overload) and job resources, including domain-specific (job control and career-related support) and boundary-spanning resources (flexible work arrangement), as the primary exposure. We hypothesize that family status, specifically entering into marriage and the acquisition of a parental role, acts as a moderator that modifies their associations with mental health. We expect these interaction effects to be most pronounced for boundary-spanning resources, which facilitate resource transfer between domains, compared to domain-specific resources.

It is also essential to consider gender differences in the potential mechanisms described above, as the intersection of work and family dynamics often varies between men and women. Recent studies of a Japanese working population found that women are more likely than men to experience work–family conflict (Tsukada, 2020), and that perceived family–work conflict is associated with work engagement among women, but not among men (Kobayashi et al., 2025). In Japan, societal expectations continue to place greater emphasis on women fulfilling roles as housewives and primary caregivers, despite the increase in female labor force participation and diversity in work and family life course patterns (McDaniel, 2015; Ruppanner, 2015). Consequently, occupying family roles like marriage and childbirth are more likely to introduce additional demands rather than resources for women, intensifying the conflict between work and family roles and potentially leading to worse mental health outcomes. In contrast, men in Japan typically maintain a work-centered identity (Shimazu, 2015), although recent trends indicate greater male involvement in housework and childcare (Goldstein-Gidoni, 2019). They continue to be viewed as primary breadwinners under a rigid gender division of labor within the household (Brinton et al., 2021; Brinton & Lee, 2016) and spend the least time on unpaid household labor among OECD countries (Miranda, 2011; OECD, 2015). As a result, men may experience less conflict between work and family roles and may even benefit from increased support provided by their spouses after marriage.

In summary, this paper will test the hypothesis that (1) family statuses modify the association of the psychosocial work environment with mental health, and (2) the modifying effect of family statuses varies between men and women. Our study makes three primary contributions to the work–family interface and mental health literatures. First, we provide a unified theoretical framework by nesting the JD–R model within the W–HR framework, specifically distinguishing between domain-specific and boundary-spanning resources to clarify how resources transfer between domains. Second, we utilize a longitudinal design to disentangle within-person changes from between-person differences, offering a robust assessment of how the work–health relationship shifts as individuals navigate interdependent life-course trajectories. Finally, we provide gendered evidence within the Japanese cultural context, identifying heterogeneous effects of work–family interactions. By highlighting these distinctions, this study offers critical insights for developing targeted workplace interventions that support well-being across significant life transitions.

## 2 Method

### 2.1 Participants and data

This study used data from the Japanese Life Course Panel Surveys (JLPS). JLPS is a nationally representative longitudinal survey of men and women living in Japan, conducted annually since 2007. JLPS collected 4800 responses in Wave 1 (2007) from two age groups: youth participants aged 20–34 and middle-aged participants aged 35–40, with response rates of 34.5% (n = 3367) and 40.4% (n = 1433), respectively (Ishida, 2013). To address attrition over time, 963 additional responses were collected in Wave 5 (2011) from participants in the same age range as the initial cohort. In addition, to compensate for the aging of the participants, 2380 new responses were collected in Wave 13 (2019) from individuals aged 20–31 at the time of the survey.

We used JLPS data from Wave 1 (2007) through Wave 14 (2020), which are currently available for secondary use from the Social Science Japan Data Archive, Center for Social Research and Data Archives, University of Tokyo. The panel data were unbalanced, and our analysis was limited to respondents who were employed at the time of each survey wave. Specifically, respondents were included in the sample for a given wave unless they were missing or unemployed. After eliminating cases with missing values, the final sample included 3531 male respondents (21316 person-year observations) and 3782 female respondents (22128 person-year observations) (see Appendix Table S1 for the distribution of respondents across waves; see Appendix Table S2 for the missing data patterns).

### 2.2 Variables

#### 2.2.1 Outcome

The mental health status was measured using the five-item version of the Mental Health Inventory (MHI-5) (Berwick et al., 1991). The MHI-5 was derived from the original 38-item Mental Health Inventory (MHI-38), developed to assess the psychological distress and well-being in the general population (Veit & Ware, 1983). The MHI-5 has been validated as an effective screening tool for mood and anxiety disorders (Rumpf et al., 2001; Cuijpers et al., 2009). This study used the Japanese version of the MHI-5, which has been validated for screening depressive symptoms in the general population in Japan (Yamazaki et al., 2005).

The respondents were asked to evaluate how often they experienced symptoms such as anxiety and depression. The items were measured on a 5-point Likert scale ranging from 1 (all of the time) to 5 (none of the time). The Cronbach’s alpha coefficient was 0.79 for the sample of this study at the baseline. The original scores of 5 to 25 were converted to scores ranging from 0 to 100 (Ware, 1996), with higher scores indicating better mental health status.

#### 2.2.2 Exposures

One set of exposures was the psychosocial work environment: work overload, job control, career-related support, and flexible work arrangements. We selected work overload as a job demands measure, whereas job control and career-related support were chosen as measures of job resources because these job characteristics are commonly found across work settings (Bakker et al., 2023; Yang et al., 2018). These measures are thus well-suited for a study targeting the general working population. We also included flexible work arrangements because they serve both as a job resource and as a boundary-spanning resource between work and family domains (Voydanoff, 2009). We use this measure to examine whether flexible work arrangements function differently from other domain-specific job resources, such as job control and career-related support.

Work overload was assessed by asking the respondents if the following statement applies to them: “I am always under the pressure of deadlines.” Respondents were asked to choose either 0 (no) or 1 (yes). Job control was assessed by asking the respondents to what extent the following statements apply to them: “I can set or change the pace of my work.” The item was measured on a 4-point Likert scale ranging from 1 (does not apply to me) to 4 (applies to me very much). These responses were dichotomized into 0 (no) and 1 (yes). Career-related support was assessed by asking the respondents if the following statement applies to them: “There are opportunities to discuss future career prospects.” Respondents were asked to choose either 0 (no) or 1 (yes). Flexible work arrangements were assessed by asking the respondents to what extent the following statement applies to them: “It is easy to adjust work arrangements in this workplace, such as shortening the working hours or taking time off to meet the needs of one's life, such as childcare, housework, or study.” This item primarily refers to the availability of flexibility, including both work scheduling and work location flexibility, rather than its actual use. The item was measured on a 4-point Likert scale ranging from 1 (does not apply to me) to 4 (applies to me very much). These responses were dichotomized into 0 (no) and 1 (yes).

Although dichotomizing psychosocial work environment variables may reduce statistical power and obscure meaningful variation, it simplifies otherwise complex interaction models. It is therefore more easily translated into organizational policies and practices. We have assessed the robustness of our findings by using continuous specifications of our variables where available.

Another set of exposures is family status, which includes marital status and parenthood. We measured marital status as a categorical variable of single, married, or divorced/widowed. Parenthood was measured as a status change variable, with a value of 0 indicating no children and 1 indicating the presence of a child. These marital status and parenthood variables capture transitions in life trajectory rather than as events. This operationalization enables us to examine the sustained psychosocial impact of occupying these roles as a spouse or parent, and how the demands and resources that accompany these roles modify the work–health relationship.

#### 2.2.3 Covariates

The socio-demographic and occupational characteristics of the participants were adjusted in the analysis. Specifically, we adjusted for age, age squared, educational attainment (i.e., college graduates or higher, junior or community college graduates, or high school graduates or lower), living standard (a five-point scale ranging from 1 = poor to 5 = affluent), maternity and childcare leave (have taken a leave or have not taken a leave), employment status (full-time, part-time, or self-employed), and occupation (professional-managerial, non-manual, or manual). We also included year dummy variables to account for time-specific heterogeneity. Educational attainment was the sole time-invariant covariate, while the other variables were time-varying covariates.

### 2.3 Statistical analysis

We used the hybrid model to simultaneously estimate the within-person and between-person effects of exposures (Allison, 2009). First, the hybrid model decomposes each time-varying predictor into person-specific mean (between-person components) and deviations from the mean (within-person components). The within-person components produce the same regression coefficients as those in the standard fixed effects models, allowing us to examine how changes in the psychosocial work environment, family status, and their interactions affect changes in mental health status while accounting for unobserved heterogeneity. Second, the hybrid model also provides estimates for time-invariant predictors and the between-person components of time-varying predictors, thereby retaining the advantage of the random effects model. We also computed Cohen’s d as a measure of individual-level effect size (Cohen, 2016). To provide a conservative estimate, we used the total standard deviation, defined as the square root of the combined within- and between-person variance components estimated from the hybrid model. Although we referenced the conventional rule of thumb (0.2: small, 0.5: medium, and 0.8: large; Cohen, 2016), this criterion may be considered overly stringent when mental health status is used as an outcome (Panjeh et al., 2023). Furthermore, we evaluated the cumulative life-course impact and population-level burden by utilizing the MHI-5 as a screening tool for depressive symptoms in the Japanese general population (Yamazaki et al., 2005). Specifically, we applied a validated cut-off of 60 points, whereby individuals scoring at or below this threshold are categorized as having moderate-to-severe depressive symptoms.

Three models were used in the analyses, each stratified by gender. First, we used a model with only psychosocial work environments as exposures to examine their association with mental health status while adjusting for socio-demographic and occupational characteristics (Model 1). Second, family status was added to the model to examine the associations of both sets of exposures with mental health status, controlling for each other (Model 2). Finally, the interaction between family status and the psychosocial work environment was included to evaluate potential modifying effects (Model 3).

All statistical analyses were conducted using Stata software (Stata Corp., College Station, TX, USA), version 19.0.

## 3 Results

Table 1 reports the descriptive statistics of the key variables in Wave 1 (2007). The mean age was 30.9 years (SD = 5.6) for men and 30.2 years (SD = 6.1) for women. For educational attainment, college graduates or higher constituted the largest proportion among men (46.6%), whereas junior or community college graduates constituted the largest proportion among women (43.1%). For family status, 47.7% of men were married, and 40.1% had children; 38.5% of women were married, and 33.4% had children. For employment status, most men were full-time workers (74.0%), while most women were either working full-time (47.4%) or part-time (47.9%).

**Table 1:** Descriptive statistics of key variables at Wave 1 (2007).

	Men (N = 1993)		Women (N = 1658)	
	Mean (SD)	n (%)	Mean (SD)	n (%)
**Socio-demographic characteristics**				
Age	30.9 (5.6)		30.2 (6.1)	
36–40 years		501 (25.1)		419 (21.0)
31–35 years		601 (30.2)		384 (19.3)
26–30 years		462 (23.2)		387 (19.4)
20–25 years		429 (21.5)		468 (23.5)
Educational attainment				
College graduate or higher		928 (46.6)		501 (30.2)
Junior or community college graduate		420 (21.1)		715 (43.1)
High school graduate or lower		645 (32.4)		442 (26.7)
Marital status				
Single		992 (49.8)		938 (56.6)
Married		951 (47.7)		638 (38.5)
Divorced/widowed		50 (2.5)		82 (4.9)
Parenthood				
Having children		800 (40.1)		554 (33.4)
No children		1193 (59.9)		1104 (66.6)
Living standard	3.01 (0.84)		3.09 (0.80)	
**Occupational characteristics**				
Employment status				
Full-time		1474 (74.0)		785 (47.4)
Part-time		361 (18.1)		794 (47.9)
Self-employed		158 (7.9)		79 (4.8)
Occupation				
Management/professional		436 (21.9)		390 (23.5)
Non-manual		787 (39.5)		1062 (64.1)
Manual		770 (38.6)		206 (12.4)
Work environment				
Work overload		438 (22.0)		209 (12.6)
Job control		1249 (62.7)		1004 (60.6)
Career-related support		729 (36.6)		770 (46.4)
Flexible work arrangement		846 (42.5)		920 (55.5)
**Mental health**	63.9 (17.6)		61.8 (18.2)	

SD: Standard deviation.

Table 2 presents the within-person effect estimates of the psychosocial work environment, family status, and interactions on mental health status among men and women. The coefficients are unstandardized and controlled for covariates. First, the work environment was significantly associated with mental health for both men and women in similar ways (Model 1). Specifically, higher work overload negatively affected mental health (Men: −3.10, CI = [−3.61, −2.60], Cohen’s *d* = −0.18; Women: −1.66, CI = [−2.24, −1.07], Cohen’s *d* = −0.10), whereas increased job control (Men: 2.57, CI = [2.09, 3.05], Cohen’s *d* = 0.15; Women: 1.27, CI = [0.83, 1.72], Cohen’s *d* = 0.08), career-related support (Men: 1.26, CI = [0.56, 1.96], Cohen’s *d* = 0.07; Women: 1.01, CI = [0.25, 1.77], Cohen’s *d* = 0.06), and flexible work arrangements (Men: 1.86, CI = [1.41, 2.31], Cohen’s *d* = 0.11; Women: 1.73, CI = [1.27, 2.19], Cohen’s *d* = 0.10) improved worker’s mental health. Second, the effect of the work environment on mental health remained significant after adding family status to the model (Model 2). Among men, entering into marriage had a significant positive association with mental health (3.38, CI = [2.39, 4.37], Cohen’s *d* = 0.20), whereas acquiring a parental role had a significant negative association with mental health (−1.05, CI = [−1.95, −0.15], Cohen’s *d* = −0.06). In contrast, these family statuses had no significant impact on women. Third, family status was allowed to interact with the work environment (Model 3). Among men, the associations of the work environment with mental health were unaffected by changes in family status. In contrast, among women, changes in family status significantly modified the association of the work environment with mental health. Specifically, entering into marriage positively affected the association between flexible work arrangement and mental health (1.60, CI = [0.18, 3.02], Cohen’s *d* = 0.10), whereas acquiring a parental role negatively affected the association between flexible work arrangement and mental health (−2.02, CI = [−3.42, −0.62], Cohen’s *d* = −0.12). We have also conducted sensitivity analysis using the original continuous specification for flexible work arrangement, and found that the results are robust.

**Table 2:** Within-person effect estimates of the psychosocial work environment, family status, and interactions on mental health status.

	Estimates (95% confidence interval) Cohen’s *d*					
	Model 1		Model 2		Model 3	
	Men	Women	Men	Women	Men	Women
**Work environment**						
Work overload	−3.10 (−3.61, −2.60)∗∗ *d* = −0.18	−1.66 (−2.24, −1.07)∗∗ *d* = −0.10	−3.08 (−3.59, −2.58)∗∗ *d* = −0.18	−1.64 (−2.23, −1.06)∗∗ *d* = −0.10	−2.75 (−3.59, −1.90)∗∗ *d* = −0.16	−2.24 (−3.17, −1.31)∗∗ *d* = −0.13
Job control	2.57 (2.09, 3.05)∗∗ *d* = 0.15	1.27 (0.83, 1.72)∗∗ *d* = 0.08	2.58 (2.10, 3.06)∗∗ *d* = 0.15	1.28 (0.83, 1.72)∗∗ *d* = 0.08	2.85 (2.10, 3.60)∗∗ *d* = 0.17	1.85 (1.11, 2.58)∗∗ *d* = 0.11
Career-related support	1.26 (0.56, 1.96)∗∗ *d* = 0.07	1.01 (0.25, 1.77)∗∗ *d* = 0.06	1.32 (0.62, 2.02)∗∗ *d* = 0.08	1.02 (0.26, 1.78)∗∗ *d* = 0.06	1.40 (0.25, 2.55)∗ *d* = 0.08	1.83 (0.60, 3.06)∗∗ *d* = 0.11
Flexible work arrangement	1.86 (1.41, 2.31)∗∗ *d* = 0.11	1.73 (1.27, 2.19)∗∗ *d* = 0.10	1.86 (1.40, 2.31)∗∗ *d* = 0.11	1.69 (1.23, 2.16)∗∗ *d* = 0.10	1.74 (1.01, 2.46)∗∗ *d* = 0.10	1.82 (1.10, 2.54)∗∗ *d* = 0.11
**Marital status**^a^						
Married			3.38 (2.39, 4.37)∗∗ *d* = 0.20	1.00 (−0.01, 2.01) *d* = 0.06	3.04 (1.41, 4.68)∗∗ *d* = 0.18	0.12 (−1.42, 1.66) *d* = 0.01
Divorced/widowed			−0.72 (−2.60, 1.15) *d* = −0.04	1.43 (−0.25, 3.10) *d* = 0.08	0.04 (−3.00, 3.08) *d* = 0.00	1.20 (−1.29, 3.68) *d* = 0.07
**Parenthood**^b^			−1.05 (−1.95, −0.15)∗ *d* = −0.06	0.35 (−0.73, 1.43) *d* = 0.02	0.26 (−1.28, 1.79) *d* = 0.02	2.13 (0.49, 3.77)∗∗ *d* = 0.13
**Interaction**						
Work overload						
× Married					−0.44 (−2.00, 1.12) *d* = −0.03	0.92 (−0.90, 2.74) *d* = 0.05
× Divorced/widowed					2.47 (−0.38, 5.32) *d* = 0.15	1.18 (−1.53, 3.88) *d* = 0.07
× Parenthood					−0.23 (−1.70, 1.23) *d* = −0.01	0.05 (−1.72, 1.82) *d* = 0.00
Job control						
× Married					0.16 (−1.33, 1.64) *d* = 0.01	−0.21 (−1.66, 1.23) *d* = −0.01
× Divorced/widowed					−0.56 (−3.33, 2.21) *d* = −0.03	0.60 (−1.52, 2.72) *d* = 0.04
× Parenthood					−0.71 (−2.15, 0.72) *d* = −0.04	−0.87 (−2.25, 0.51) *d* = −0.05
Career-related support						
× Married					−1.44 (−3.63, 0.76) *d* = −0.09	−1.82 (−4.34, 0.70) *d* = −0.11
× Divorced/widowed					−2.14 (−6.58, 2.31) *d* = −0.13	−1.77 (−5.54, 1.99) *d* = −0.11
× Parenthood					1.61 (−0.46, 3.68) *d* = 0.10	0.63 (−1.83, 3.09) *d* = 0.04
Flexible work arrangement						
× Married					0.84 (−0.57, 2.24) *d* = 0.05	1.60 (0.18, 3.02)∗ *d* = 0.10
× Divorced/widowed					−1.52 (−4.14, 1.11) *d* = −0.09	−0.12 (−2.30, 2.06) *d* = −0.01
× Parenthood					−0.67 (−2.02, 0.68) *d* = −0.04	−2.02 (−3.42, −0.62)∗∗ *d* = −0.12

Estimates of covariates were omitted for simplicity (See Table S3 for the estimates of covariates).

∗p < 0.05, ∗∗p < 0.01.

a Reference category: single.

b ^Reference category: no children.^

**Fig. 2:**
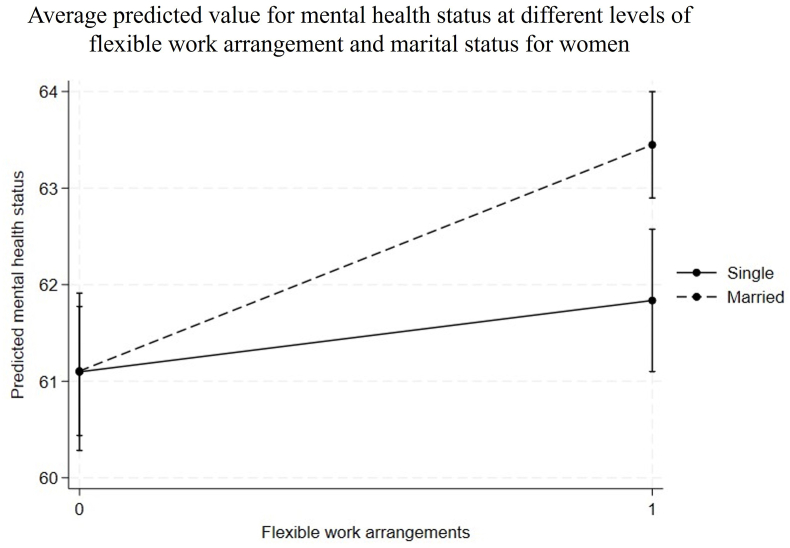
Average predicted value for mental health status at different levels of flexible work arrangement and marital status for women.

**Fig. 3:**
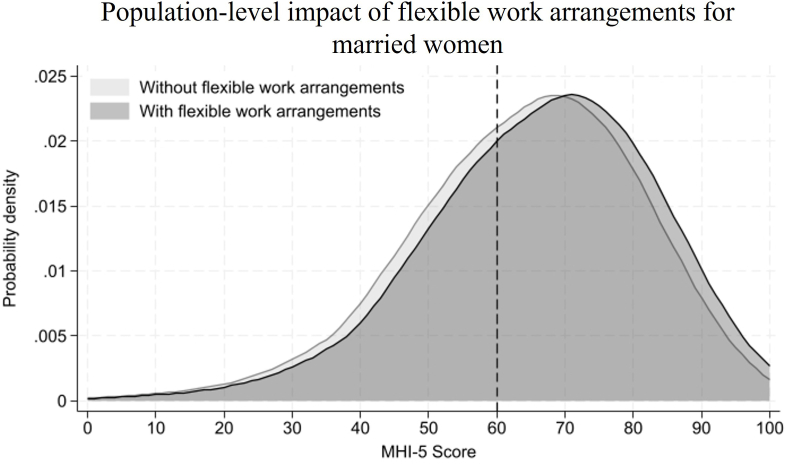
Population-level impact of flexible work arrangements for married women.

Fig. 2, Fig. 3 graphically illustrate the modifying effects of entering into marriage on the within-person associations between flexible work arrangements and mental health among women, as well as its population-level impact. Fig. 2 presents the average predicted value for mental health status at different levels of flexible work arrangement and marital status for women. The two lines in Fig. 2 represent the same individuals, first when they were single and then when they were married. The slopes of these lines indicate the average marginal effect of flexible work arrangements on mental health. Entering into marriage had a positive modifying effect: the mental health benefit of flexible work arrangements was significant only after the women entered into marriage (2.35, CI = [1.58, 3.11], Cohen’s *d* = 0.13), while it was non-significant when they were single (0.75, CI = [−0.19, 1.68], Cohen’s *d* = 0.04).

To illustrate the population-level impact of this interaction effect, Fig. 3 presents kernel density plots of the MHI-5 score among women after entering into marriage, with and without flexible work arrangements. The population at risk, or those scoring 60 or lower, constitutes 43.2% of the population among women without flexible work arrangements. Access to flexible work arrangements is associated with a 2.35-point improvement in the MHI-5 score, reducing the population at risk to 31.4%. This shift results in 11.9% of the population crossing the clinical threshold for moderate-to-severe depression (corresponding to the approximate difference in the area under the curves below the 60 cut-off), representing a 27.4% relative reduction in the prevalence of moderate-to-severe depressive symptoms in this group.

**Fig. 4:**
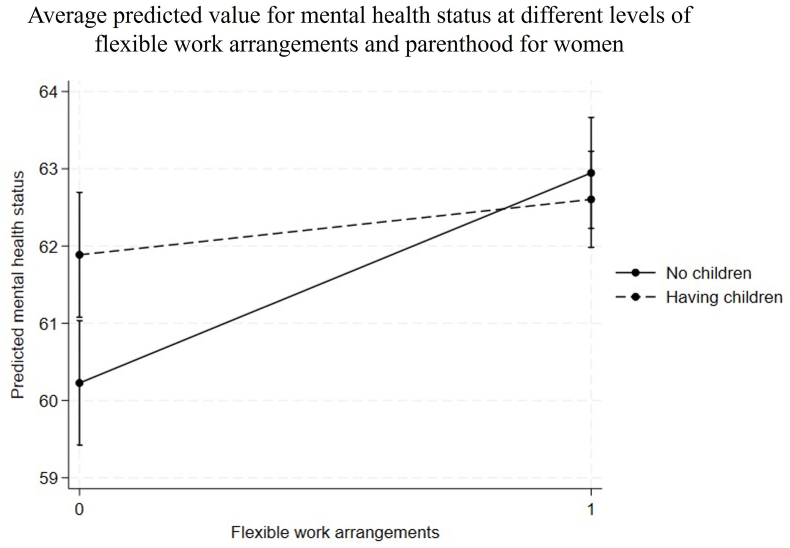
Average predicted value for mental health status at different levels of flexible work arrangements and parenthood for women.

**Fig. 5:**
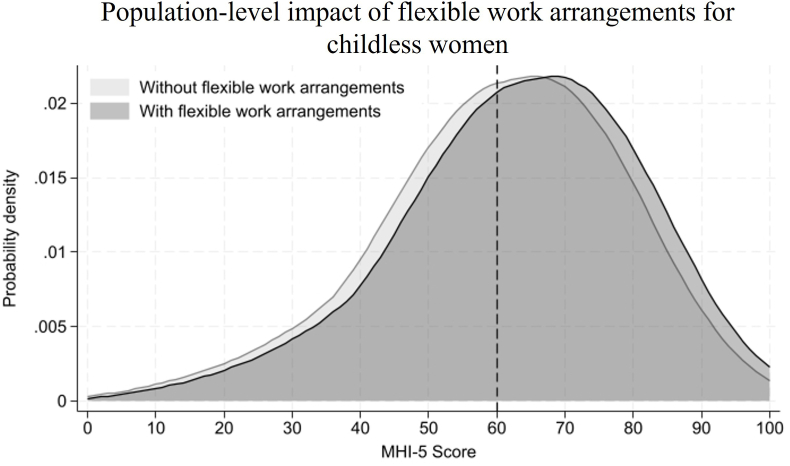
Population-level impact of flexible work arrangements for childless women.

Fig. 4, Fig. 5 graphically illustrate the modifying effects of acquiring a parental role on the within-person associations between flexible work arrangements and mental health among women, as well as its population-level impact. Fig. 4 presents the average predicted value for mental health status at different levels of flexible work arrangements and parenthood for women. The two lines in Fig. 4 represent the same individuals, first when they were childless and then when they had at least one child. The slopes of these lines indicate the average marginal effect of flexible work arrangements on mental health. The acquisition of a parental role had a negative modifying effect, such that the mental health benefit of flexible work arrangements was non-significant after childbirth (0.71, CI = [−0.11, 1.54], Cohen’s *d* = 0.04), while it was significant when the women were childless (2.73, CI = [1.87, 3.59], Cohen’s *d* = 0.15).

To illustrate the population-level impact of this interaction effect, Fig. 5 presents kernel density plots of the MHI-5 score among women after acquiring a parental role, with and without a flexible work arrangement. The population at risk, or those scoring 60 or lower, constitutes 51.3% of the population among women without flexible work arrangements. Access to flexible work arrangements is associated with a 2.73-point improvement in the MHI-5 score, reducing the population at risk to 39.7%. This shift results in 11.6% of the population crossing the clinical threshold for moderate-to-severe depression (corresponding to the approximate difference in the area under the curves below the 60 cut-off), representing a 22.6% relative reduction in the prevalence of moderate-to-severe depressive symptoms in this group.

Table 3 presents the between-person effect estimates of the psychosocial work environment, family status, and their interactions on mental health among men and women. The coefficients are unstandardized and controlled for covariates. First, the work environment was significantly associated with mental health for both men and women in a similar way, except for job control (Model 1). That is, higher work overload was associated with lower mental health (Men: −6.95, CI = [−8.40, −5.50], Cohen’s *d* = −0.41; Women: −6.36, CI = [−8.08, −4.64], Cohen’s *d* = −0.38), whereas career-related support (Men: 2.27, CI = [0.09, 4.44], Cohen’s *d* = 0.13; Women: 2.55, CI = [0.17, 4.93], Cohen’s *d* = 0.15) and flexible work arrangements (Men: 3.71, CI = [2.38, 5.04], Cohen’s *d* = 0.22; Women: 3.49, CI = [2.12, 4.85], Cohen’s *d* = 0.21) were associated with higher mental health. Job control was positively associated with mental health only among men (3.38, CI = [2.01, 4.75], Cohen’s *d* = 0.20). Second, these associations between work environment and mental health persisted after adding family statuses to the model (Model 2). For both men and women, being married was significantly associated with better mental health status compared to being single (Men: 3.98, CI = [2.11, 5.85], Cohen’s *d* = 0.24; Women: 3.32, CI = [1.55, 5.09], Cohen’s *d* = 0.20). Third, family status was allowed to interact with the work environment (Model 3). However, no modifying effects of family status on the associations between work environment and mental health were observed for either men or women.

**Table 3:** Between-person effect estimates of the psychosocial work environment, family status, and interactions on mental health status.

	Estimates (95% confidence interval) Cohen’s *d*					
	Model 1		Model 2		Model 3	
	Men	Women	Men	Women	Men	Women
**Work environment**						
Work overload	−6.95 (−8.40, −5.50)∗∗ *d* = −0.41	−6.36 (−8.08, −4.64)∗∗ *d* = −0.38	−7.02 (−8.46, −5.57)∗∗ *d* = −0.42	−6.30 (−8.02, −4.59)∗∗ *d* = −0.37	−7.74 (−9.87, −5.61)∗∗ *d* = −0.46	−5.96 (−8.35, −3.56)∗∗ *d* = −0.36
Job control	3.38 (2.01, 4.75)∗∗ *d* = 0.20	1.01 (−0.25, 2.26) *d* = 0.06	3.25 (1.88, 4.61)∗∗ *d* = 0.19	1.09 (−0.17, 2.34) *d* = 0.06	3.55 (1.71, 5.39)∗∗ *d* = 0.21	1.53 (−0.23, 3.30) *d* = 0.09
Career-related support	2.27 (0.09, 4.44)∗ *d* = 0.13	2.55 (0.17, 4.93)∗ *d* = 0.15	2.28 (0.11, 4.45)∗ *d* = 0.14	2.69 (0.31, 5.06)∗ *d* = 0.16	1.83 (−1.25, 4.92) *d* = 0.11	2.77 (−0.40, 5.94) *d* = 0.17
Flexible work arrangement	3.71 (2.38, 5.04)∗∗ *d* = 0.22	3.49 (2.12, 4.85)∗∗ *d* = 0.21	3.64 (2.32, 4.97)∗∗ *d* = 0.22	2.95 (1.55, 4.34)∗∗ *d* = 0.18	3.53 (1.67, 5.39)∗∗ *d* = 0.21	1.98 (0.15, 3.80)∗ *d* = 0.12
**Marital status**^a^						
Married			3.98 (2.11, 5.85)∗∗ *d* = 0.24	3.32 (1.55, 5.09)∗∗ *d* = 0.20	5.88 (1.44, 10.3)∗ *d* = 0.35	4.25 (0.32, 8.18)∗ *d* = 0.25
Divorced/widowed			−0.46 (−3.86, 2.93) *d* = −0.03	0.58 (−2.02, 3.17) *d* = 0.03	−3.18 (−10.9, 4.55) *d* = −0.19	−1.29 (−7.61, 5.03) *d* = −0.08
**Parenthood**^b^			−1.32 (−3.13, 0.49) *d* = −0.08	−0.26 (−1.94, 1.43) *d* = −0.02	−3.50 (−8.06, 1.06) *d* = −0.21	−2.36 (−6.47, 1.76) *d* = −0.14
**Interaction**						
Work overload						
× Married					1.44 (−3.69, 6.57) *d* = 0.09	−3.38 (−9.22, 2.46) *d* = −0.20
× Divorced/widowed					8.31 (−2.24, 18.9) *d* = 0.49	−3.94 (−12.9, 5.01) *d* = −0.24
× Parenthood					−0.62 (−5.78, 4.54) *d* = −0.04	3.74 (−2.17, 9.66) *d* = 0.22
Job control						
× Married					−2.02 (−7.15, 3.10) *d* = −0.12	−0.84 (−5.19, 3.52) *d* = −0.05
× Divorced/widowed					−1.13 (−10.8, 8.51) *d* = −0.08	3.13 (−4.00, 10.3) *d* = 0.19
× Parenthood					1.66 (−3.62, 6.95) *d* = 0.10	−0.74 (−5.13, 3.65) *d* = −0.04
Career-related support						
× Married					0.98 (−7.79, 9.74) *d* = 0.06	−2.44 (−11.3, 6.46) *d* = −0.15
× Divorced/widowed					−0.76 (−14.9, 13.4) *d* = −0.05	4.57 (−8.50, 17.6) *d* = 0.27
× Parenthood					0.09 (−8.77, 8.94) *d* = 0.01	2.17 (−6.80, 11.1) *d* = 0.13
Flexible work arrangement						
× Married					−2.47 (−7.38, 2.44) *d* = −0.15	0.47 (−4.03, 4.98) *d* = 0.03
× Divorced/widowed					3.69 (−5.36, 12.7) *d* = 0.22	0.36 (−6.52, 7.24) *d* = 0.02
× Parenthood					3.00 (−1.98, 7.98) *d* = 0.18	2.62 (−2.04, 7.28) *d* = 0.16

Estimates of covariates were omitted for simplicity (See Table S4 for the estimates of covariates).

∗p < 0.05, ∗∗p < 0.01.

a Reference category: single.

b Reference category: no children.

## 4 Discussion

### 4.1 Interpretations

In summary, our findings indicated that, at the within-person level, changes in family status significantly modified the association of the psychosocial work environment with mental health among women. Specifically, entering into marriage and acquiring a parental role altered the within-person associations between flexible work arrangements and mental health. Among men, by contrast, the mental health impact of the psychosocial work environment remained consistent regardless of changes in family status. At the between-person level, we found no evidence of modifying effects of family status differences on the associations between psychosocial work environment and mental health.

First, there was a positive interaction effect between flexible work arrangements and women’s marriage: the mental health benefits of flexible work arrangements became significant only after women entered into marriage. In Japan, the gender division of household labor remains persistent, with women expected to take on greater responsibility for housework after marriage, even in dual-earner couples (Brinton et al., 2021; McDaniel, 2015; Ruppanner, 2015). This new role as a housewife may enable women to make more effective use of flexible work arrangements in the workplace, which in turn helps them manage their expanded roles and enhances their psychological well-being (Talley et al., 2012).

Second, we observed a negative interaction between flexible work arrangements and women’s parenthood: the mental health benefits of flexible work arrangements disappeared once women became mothers. Past studies have reported mixed evidence regarding the impact of flexible work arrangements on parents’ mental health (Hokke et al., 2021). A plausible explanation for our findings is that flexible work arrangements may be underutilized or used sub-optimally by women transitioning into motherhood, preventing these arrangements from contributing to improvements in their mental health. Several mechanisms potentially underlie this explanation. First, working mothers may rely on maternity and childcare leave to cope with the demands of childbirth and to improve their mental health, or resources they did not have when they were childless. In Japan, most women who continue staying in the workforce take maternity and childcare leave when they become pregnant or give birth (Mun & Brinton, 2015). During these care-intensive years, parental leave may serve as the primary resource to offset the demands of child caring, reducing the relative importance and potential benefits of flexible work arrangements. Second, mothers may rely on informal work accommodations regardless of the availability of formal flexible work arrangements. In other words, mothers may manage childcare needs through self-directed day-to-day adjustments, rather than using the formal arrangements provided by employers (Hokke et al., 2021). This pattern is especially likely in Japan, where working mothers are expected to simultaneously comply with the household gender-role norm that demands intensive mothering and the ideal-worker norm that emphasizes a full commitment to the workplace (Brinton & Mun, 2016). The work intensification perspective offers another plausible explanation for these findings (Niazi et al., 2024). In Japan, mothers often face an exceptionally heavy caregiving burden, synonymous with role overload, compounded by the fact that fathers rarely take parental leave (Fuwa, 2021). Under such circumstances, workplace flexibility may do little to alleviate the intensive demands placed on mothers. Instead, by blurring the work–home boundary, flexibility might inadvertently exacerbate the strain of intensive mothering as mothers use the additional resources these arrangements provide to meet escalating domestic demands (Kubicek & Tement, 2016). In this context, flexibility may cease to function as a resource that improves mental health, serving instead as a mechanism for further absorbing an individual’s remaining time into the family domain.

Third, while individual-level Cohen’s *d* values for within-person interaction effects among women appear modest under traditional criteria (e.g., *d* = 0.2), small shifts in a population’s mean well-being can result in a significant proportion of at-risk individuals crossing critical clinical thresholds. Specifically, the 2.35-point benefit of flexible work arrangements for married women translates into 27.4% relative reduction in the prevalence of moderate-to-severe depressive symptoms. Similarly, the 2.73-point benefit of flexibility for childless women leads to a 22.6% relative reduction. These findings underscore that workplace flexibility could be a critical resource for primary prevention. In the contemporary Japanese context, where women frequently navigate the tension between the ideal-worker norm and caregiving demands (Brinton & Mun, 2016), and where companies are trying to implement flexibility policies (Goldstein-Gidoni, 2025), this study provides evidence that targeted flexibility policies can substantially improve the mental health of the population across their life course.

Fourth, this study identified gender differences in how family statuses modify the mental health effects of flexible work arrangements, whereas domain-specific job resources, such as job control and career-related support, did not interact with family domains for either gender. Although flexible work arrangements are boundary-spanning job resources that can extend into the family domain (Voydanoff, 2009), male workers may use them primarily as job resources within the work domain, even when their family status changes. One reason is that, even after entering into marriage or acquiring a parental role, men’s primary role often remains work-centered (Shimazu, 2015). Thus, men may have less need to utilize flexible work arrangements to manage the work–family interface compared to women. Another reason is that male workers often face stronger pressure to adhere to the ideal-worker norm than their female counterparts, which may discourage them from using flexible work arrangements to address housework or childcare demands (Behson et al., 2018). Instead, flexible work arrangements might be used solely as job resources to directly or indirectly meet the demands of the workplace. Beyond these role-centered interpretations, methodological factors may have also contributed to these results. First, while our measure captures the availability of flexible work arrangements, actual utilization may vary by gender. In Japan, men often refrain from using available flexibility for family purposes to avoid potential career penalties or flexibility stigma (Ferdous et al., 2022; Goldstein-Gidoni, 2025). If the resource is available but not utilized for family requirements, its potential to modify the relationship between family transitions and mental health would be diminished. Second, men may be more likely to over-report mental health status due to adherence to traditional masculine norms (Branney & White, 2008), which could lead to a reduced variance in the MHI-5 scores. Table 1 shows that men have higher MHI-5 scores on average and exhibit less variation. Such measurement insensitivity might limit our ability to detect subtle interaction effects within the male workers.

Fifth, this study did not find any modifying effects of family status differences on the associations between the psychosocial work environment and mental health at the between-person level. In other words, life transitions such as marriage and parenthood modified the relationship between flexible work arrangements and mental health only within individuals over time, not across individuals. A similar pattern was reported by Zhao et al. (2021), who found within-person effects but no between-person differences. In their longitudinal analysis of working parents in Australia, fathers who transitioned into irregular shifts experienced a decline in marital relationship quality, whereas no significant differences were observed between those who consistently worked standard versus nonstandard schedules. One plausible explanation that applies to the present study is that between-person estimates average across individuals with diverse and stable life circumstances, which can obscure effects that become evident only when individuals experience meaningful life transitions.

### 4.2 Limitations

Several limitations of the present study need to be noted. First, this study focused exclusively on the working population in Japan, where a substantial proportion of women leave the labor market when they give birth to their first child (Mun & Brinton, 2015). One major reason for women’s labor market exit is the challenge of meeting the ideal-worker norm for undiluted commitment to the workplace while simultaneously bearing primary responsibility for the family (Brinton & Mun, 2016). Thus, the findings from this study may not be generalizable to countries with different demographic, socioeconomic, and cultural contexts.

Second, this study relied on a single-item measure for overall flexibility at the workplace, limiting our ability to identify the mechanisms underlying the observed modifying effects. In particular, the item used in this study reflects multiple aspects of flexible work arrangements, including work scheduling flexibility (e.g., flextime), work location flexibility (e.g., remote work), and taking leave with or without pay. Additionally, the item measures availability rather than actual usage, which is known to have distinctive impacts on outcomes (Hokke et al., 2021). Future studies should differentiate among specific components of flexible work arrangements to provide a more nuanced understanding of how family statuses may alter the mental health impact of flexible work arrangements for men and women.

Third, this study mainly focused on individual changes over the life course (i.e., age effects). Studies have pointed out that younger cohorts, especially men, place greater value on workplace accommodations, including flexibility (Behson et al., 2018), a trend also observed in Japan (Goldstein-Gidoni, 2019). These cohort differences in preferences and values regarding the work–family interface imply that the interaction between work and family domains may vary between younger and older generations. Therefore, future research employing a life course perspective should incorporate both chronological and historical time into the analysis to further elaborate on the interactions between work and family domains across different cohorts.

Fourth, while this study uses a life-course perspective to examine within-person changes, marriage and parenthood were operationalized as time-varying status indicators rather than acute transition events. Consequently, our estimates reflect the average difference in mental health status between periods of occupying different social roles (e.g., being single vs. being married) rather than the immediate impact of the transition event itself. Future research could disentangle the acute effects of entering these roles from the long-term impacts of role-occupancy.

### 4.3 Implications

Despite these limitations, this study provides both theoretical and practical implications. First, this study employed the life course perspective to disentangle how work and family circumstances interact to shape the mental health of male and female workers at both within- and between-individual levels. Our findings underscore that the boundary-spanning aspect of the psychosocial work environment is more likely to interact with family status than domain-specific job demands and resources. These findings demonstrate the theoretical utility of nesting the JD–R model within the W–HR framework, particularly by highlighting the distinct roles of job resources. In particular, job demands and resources may not be the only relevant categories when considered together with the family domain (Tsukada, 2020). Instead, some job characteristics may function as boundary-spanning demands or resources, bridging work and family domains (Voydanoff, 2009). Second, past studies on work–family interactions have attempted to categorize countries into broader regions, such as Confucian Asia, encompassing Japan, Korea, and China (Cho & Choi, 2018). While this perspective of shared culture is valuable, the present study’s findings cannot be fully explained without attention to Japan’s distinctive institutional and social context, underscoring the necessity of a cross-national perspective for understanding work–family interactions.

From a practical perspective, the observed gender heterogeneity in interaction effects suggests that workplace interventions aimed at promoting mental health may yield differing outcomes for men and women, especially during significant life transitions. A universal intervention strategy targeting both male and female workers equally risks overlooking the distinct needs of certain women. This study found that flexible work arrangements are a stronger predictor of mental health for women who entered into marriage. The substantial clinical significance of these findings, evidenced by an absolute risk reduction of 11.9% in the prevalence of moderate-to-severe depressive symptoms, suggests that targeted flexibility policies are a highly efficient primary prevention tool for improving population health. Enhancing workplace flexibility for married women with family responsibilities could therefore serve as a targeted intervention to help close their health gap with male workers. However, the mental health benefits of flexible work arrangements decline for women acquiring a parental role, suggesting that flexibility alone is insufficient. Supplementary support measures may be necessary for women with child-caring roles who face elevated mental health risks in the workplace. By recognizing and addressing the specific challenges and circumstances women encounter during these transitional periods, organizations can implement targeted interventions that provide direct support where it is most needed.

### 4.4 Conclusion

The present study examined whether family status modifies the association of the psychosocial work environment with mental health among men and women in Japan across the life course. Drawing on the W–HR and JD–R models, we focused on domain-specific job demands and resources (work overload, job control, and career-related support) as well as flexible work arrangements as boundary-spanning resources. Using the hybrid model, we found that changes in family status significantly modified the association of the psychosocial work environment with mental health for women at the within-person level. Specifically, entering into marriage and transitioning to parenthood modified the within-person associations between flexible work arrangements and mental health for women. For men, however, the mental health impact of the psychosocial work environment remained stable regardless of changes in family status. At the between-person level, we found no modifying effect of family status differences on the associations between psychosocial work environment and mental health. These findings suggest that workplace flexibility provides substantial public health gains through primary prevention for women navigating gendered life-course transitions. The heterogeneity in the modifying effect by gender likely reflects prevailing household gender-role norms that expect women to take on roles as homemakers and primary caregivers, while men remain work-centered breadwinners. These divergent role expectations shape how men and women utilize flexible work arrangements as a resource. Future research should differentiate specific components of flexible work arrangements to further elaborate on the mechanisms underlying these modifying effects.

## CRediT authorship contribution statement

**Nobutada Yokouchi:** Writing – original draft, Methodology, Formal analysis, Data curation, Conceptualization. **Hiroshi Ishida:** Writing – review & editing, Supervision, Methodology, Funding acquisition, Conceptualization.

## Ethical statement

This study is secondary analysis of the Japanese Life Course Panel Surveys which is approved by the Institutional Research Ethics Review Board at the Institute of Social Science, University of Tokyo.

## Declaration of interest statement

No potential conflict of interest was reported by the authors.

## Data Availability

Data used in this study are currently available for secondary use from the Social Science Japan Data Archive, Center for Social Research and Data Archives, University of Tokyo.
