# Supplementary material for: How work and family dynamics shape mental health across the life course: Insights from a longitudinal study in Japan
Source: SSM Popul Health. 2026 Apr 3;34:101920. doi: 10.1016/j.ssmph.2026.101920 (PMC13089081; doi:10.1016/j.ssmph.2026.101920)
Supplement: Multimedia component 1 [file mmc1.docx]

# **Appendices**

**Table S1**

Distribution of respondents across waves

|  | Men (N = 1,993) | | | |  | Women (N = 1,658) | | | |
| --- | --- | --- | --- | --- | --- | --- | --- | --- | --- |
| Year | Continuation Sample (%**^a^**) | Additional sample (%**^b^**) | Refresh sample (%**^c^**) | Total |  | Continuation Sample (%**^a^**) | Additional sample (%**^b^**) | Refresh sample (%**^c^**) | Total |
| 2007 | 1,993 (NA) | 0 | 0 | 1,993 |  | 1,658 (NA) | 0 | 0 | 1,658 |
| 2008 | 1,655 (83.0) | 0 | 0 | 1,655 |  | 1,432 (86.4) | 0 | 0 | 1,432 |
| 2009 | 1,474 (89.1) | 0 | 0 | 1,474 |  | 1,327 (92.7) | 0 | 0 | 1,327 |
| 2010 | 1,217 (82.6) | 0 | 0 | 1,217 |  | 1,227 (92.5) | 0 | 0 | 1,227 |
| 2011 | 1,308 (107.5) | 356 (NA) | 0 | 1,664 |  | 1,285 (104.7) | 354 (NA) | 0 | 1,639 |
| 2012 | 1,248 (95.4) | 259 (72.8) | 0 | 1,507 |  | 1,238 (96.3) | 290 (81.9) | 0 | 1,528 |
| 2013 | 1,217 (97.5) | 249 (96.1) | 0 | 1,466 |  | 1,238 (100.0) | 289 (99.7) | 0 | 1,527 |
| 2014 | 1,157 (95.1) | 219 (88.0) | 0 | 1,376 |  | 1,201 (97.0) | 279 (96.5) | 0 | 1,480 |
| 2015 | 1,121 (96.9) | 204 (93.2) | 0 | 1,325 |  | 1,150 (95.8) | 263 (94.3) | 0 | 1,413 |
| 2016 | 1,117 (99.6) | 214 (104.9) | 0 | 1,331 |  | 1,181 (102.7) | 298 (113.3) | 0 | 1,479 |
| 2017 | 1,016 (91.0) | 200 (93.5) | 0 | 1,216 |  | 1,058 (89.6) | 254 (85.2) | 0 | 1,312 |
| 2018 | 1,058 (104.1) | 204 (102.0) | 0 | 1,262 |  | 1,175 (111.1) | 285 (112.2) | 0 | 1,460 |
| 2019 | 1,020 (96.4) | 203 (99.5) | 801 (NA) | 2,024 |  | 1,133 (96.4) | 275 (96.5) | 975 (NA) | 2,383 |
| 2020 | 1,016 (99.6) | 202 (99.5) | 588 (73.4) | 1,806 |  | 1,170 (103.3) | 293 (106.5) | 800 (82.1) | 2,263 |
| Total | 17,617 | 2,310 | 1,389 | 21,316 |  | 17,473 | 2,880 | 1,775 | 22,128 |

^a^ Retention rate based on consecutive surveys for the continuation sample from 2007

^b^ Retention rate based on consecutive surveys for the additional sample from 2011

^c^ Retention rate based on consecutive surveys for the refresh sample from 2019

**Table S2**

Missing data pattern

|  | Person-year observations (%) | |
| --- | --- | --- |
| Number of missing values**^a^** | Men | Women |
| 0 | 21,316 (40.0) | 22,128 (42.0) |
| 1 | 1,446 (2.7) | 1,413 (2.7) |
| 2 | 266 (0.5) | 208 (0.4) |
| 3 | 78 (0.2) | 69 (0.1) |
| 4 | 122 (0.2) | 117 (0.2) |
| 5 | 38 (0.1) | 40 (0.1) |
| 6 | 16 (0.0) | 12 (0.0) |
| 7 | 10 (0.0) | 1 (0.0) |
| 8 | 2 (0.0) | 2 (0.0) |
| 9 | 2 (0.0) | 1 (0.0) |
| 10 | 1,934 (3.6) | 1,503 (2.9) |
| 11 | 17,094 (32.1) | 14,565 (27.7) |
| 12 | 10,955 (20.6) | 12,583 (23.9) |
| Total | 53,279 | 52,642 |

^a^ Including 12 analytical variables (outcomes, exposures, and covariates) except for gender, age, and age squared, which had no missing values

**Table S3**

Within-person effect estimates of the covariates.

|  | Estimates (95% confidence interval)  Cohen’s *d* | | | | | |
| --- | --- | --- | --- | --- | --- | --- |
|  | Model 1 | | Model 2 | | Model 3 | |
|  | Men | Women | Men | Women | Men | Women |
| Age | 0.25 (−0.08, 0.58)  *d* = 0.01 | 0.34 (0.04, 0.64)^*^  *d* = 0.02 | 0.02 (−0.33, 0.37)  *d* = 0.00 | 0.17 (−0.17, 0.51)  *d* = 0.01 | −0.04 (−0.39, 0.31)  *d* = −0.00 | 0.16 (−0.17, 0.50)  *d* = 0.01 |
| Age squared | −0.01 (−0.01, −0.00)^*^  *d* = −0.00 | −0.01 (−0.01, 0.00)^*^  *d* = −0.00 | −0.00 (−0.01, 0.00)  *d* = −0.00 | −0.00 (−0.01, 0.00)  *d* = −0.00 | −0.00 (−0.01, 0.00)  *d* = −0.00 | −0.00 (−0.01, 0.00)  *d* = −0.00 |
| Educational attainment^c^ |  |  |  |  |  |  |
| Junior or community college graduates | NA | NA | NA | NA | NA | NA |
| College graduates or higher | NA | NA | NA | NA | NA | NA |
| Living standard | 2.08 (1.73, 2.42)^**^  *d* = 0.12 | 1.72 (1.37, 2.07)^**^  *d* = 0.10 | 2.08 (1.73, 2.42)^**^  *d* = 0.12 | 1.72 (1.37, 2.07)^**^  *d* = 0.10 | 2.08 (1.73, 2.43)^**^  *d* = 0.12 | 1.72 (1.37, 2.07)^**^  *d* = 0.10 |
| Maternity and childcare leave^d^ | 2.16 (−1.10, 5.42)  *d* = 0.13 | 1.52 (0.54, 2.51)^**^  *d* = 0.09 | 1.71 (−1.55, 4.97)  *d* = 0.10 | 1.31 (0.31, 2.31)^**^  *d* = 0.08 | 1.70 (−1.57, 4.97)  *d* = 0.10 | 1.23 (0.18, 2.27)^**^  *d* = 0.07 |
| Employment status^e^ |  |  |  |  |  |  |
| Part-time | 0.22 (−0.71, 1.15)  *d* = 0.01 | 1.92 (1.25, 2.59)^**^  *d* = 0.11 | 0.44 (−0.48, 1.37)  *d* = 0.03 | 1.84 (1.16, 2.52)^**^  *d* = 0.11 | 0.49 (−0.44, 1.42)  *d* = 0.03 | 1.82 (1.14, 2.50)^**^  *d* = 0.11 |
| Self-employed | −0.53 (−1.82, 0.76)  *d* = −0.03 | 1.77 (0.35, 3.18)^*^  *d* = 0.11 | −0.54 (−1.83, 0.75)  *d* = −0.03 | 1.69 (0.27, 3.11)^**^  *d* = 0.10 | −0.55 (−1.84, 0.75)  *d* = −0.03 | 1.79 (0.37, 3.21)^**^  *d* = 0.11 |
| Occupation^f^ |  |  |  |  |  |  |
| Professional-managerial | −0.96 (−2.12, −0.19)  *d* = −0.06 | −1.05 (−2.31, −0.20)  *d* = −0.06 | −1.02 (−2.17, −0.13)^**^  *d* = −0.06 | −1.00 (−2.26, −0.25)^**^  *d* = −0.06 | −1.00 (−2.15, −0.15)^**^  *d* = −0.06 | −1.05 (−2.30, −0.21)^**^  *d* = −0.06 |
| Non-manual | −0.59 (−1.53, −0.36)  *d* = −0.03 | −1.41 (−2.33, −0.48)^**^  *d* = −0.08 | −0.63 (−1.57, −0.31)^**^  *d* = −0.04 | −1.40 (−2.32, −0.48)^**^  *d* = −0.08 | −0.64 (−1.58, 0.31)  *d* = −0.04 | −1.43 (−2.35, −0.50)^**^  *d* = −0.09 |
| Year dummy^g^ |  |  |  |  |  |  |
| 2008 | −0.72 (−1.51, −0.06)  *d* = −0.04 | −1.10 (−1.94, −0.27)^*^  *d* = −0.07 | −0.74 (−1.52, −0.04)^**^  *d* = −0.04 | −1.09 (−1.93, −0.26)^**^  *d* = −0.06 | −0.71 (−1.49, 0.07)  *d* = −0.04 | −1.09 (−1.92, −0.26)^**^  *d* = −0.07 |
| 2009 | −1.00 (−1.80, −0.21)^*^  *d* = −0.06 | −0.45 (−1.29, 0.38)  *d* = −0.03 | −1.00 (−1.80, −0.21)^**^  *d* = −0.06 | −0.42 (−1.26, 0.41)  *d* = −0.03 | −0.98 (−1.77, −0.19)^**^  *d* = −0.06 | −0.40 (−1.23, 0.44)  *d* = −0.02 |
| 2010 | −2.62 (−3.45, −1.79)^**^  *d* = −0.16 | −2.10 (−2.94, −1.26)^**^  *d* = −0.13 | −2.63 (−3.47, −1.80)^**^  *d* = −0.16 | −2.08 (−2.92, −1.23)^**^  *d* = −0.12 | −2.60 (−3.43, −1.77)^**^  *d* = −0.15 | −2.06 (−2.90, −1.22)^**^  *d* = −0.12 |
| 2011 | −0.92 (−1.67, −0.16)^*^  *d* = −0.05 | 0.14 (−0.62, 0.91)  *d* = 0.01 | −0.91 (−1.67, −0.16)^**^  *d* = −0.05 | 0.17 (−0.60, 0.94)  *d* = 0.01 | −0.88 (−1.63, −0.12)^**^  *d* = −0.05 | 0.17 (−0.59, 0.94)  *d* = 0.01 |
| 2012 | −0.17 (−0.93, 0.59)  *d* = −0.01 | 0.15 (−0.61, 0.91)  *d* = 0.01 | −0.18 (−0.94, 0.58)  *d* = −0.01 | 0.18 (−0.58, 0.94)  *d* = 0.01 | −0.13 (−0.89, 0.63)  *d* = −0.01 | 0.18 (−0.58, 0.95)  *d* = 0.01 |
| 2013 | −1.02 (−1.78, −0.25)^*^  *d* = −0.06 | −0.40 (−1.16, −0.35)  *d* = −0.02 | −0.98 (−1.74, −0.22)^**^  *d* = −0.06 | −0.37 (−1.12, 0.38)  *d* = −0.02 | −0.95 (−1.72, −0.19)^**^  *d* = −0.06 | −0.36 (−1.11, 0.39)  *d* = −0.02 |
| 2014 | −0.36 (−1.13, 0.42)  *d* = −0.02 | 0.00 (−0.75, 0.76)  *d* = 0.00 | −0.32 (−1.10, 0.46)  *d* = −0.02 | 0.03 (−0.72, 0.79)  *d* = 0.00 | −0.30 (−1.08, 0.48)  *d* = −0.02 | 0.03 (−0.72, 0.79)  *d* = 0.00 |
| 2015 | −0.00 (−0.80, 0.79)  *d* = −0.00 | −0.88 (−1.64, −0.11)^*^  *d* = −0.05 | 0.00 (−0.79, 0.79)  *d* = 0.00 | −0.85 (−1.62, −0.09)^**^  *d* = −0.05 | 0.03 (−0.76, 0.82)  *d* = 0.00 | −0.86 (−1.62, −0.09)^**^  *d* = −0.05 |
| 2016 | −0.43 (−1.23, 0.37)  *d* = −0.03 | −0.84 (−1.59, −0.09)^*^  *d* = −0.05 | −0.46 (−1.26, 0.34)  *d* = −0.03 | −0.82 (−1.58, −0.07)^**^  *d* = −0.05 | −0.43 (−1.23, 0.37)  *d* = −0.03 | −0.84 (−1.60, −0.09)^**^  *d* = −0.05 |
| 2017 | −0.69 (−1.53, 0.14)  *d* = −0.04 | −1.12 (−1.91, −0.34)^*^  *d* = −0.07 | −0.70 (−1.53, 0.14)  *d* = −0.04 | −1.11 (−1.89, −0.32)^**^  *d* = −0.07 | −0.69 (−1.52, 0.14)  *d* = −0.04 | −1.11 (−1.90, −0.32)^**^  *d* = −0.07 |
| 2018 | 0.11 (−0.73, 0.96)  *d* = 0.01 | 0.27 (−0.50, 1.04)  *d* = 0.02 | 0.13 (−0.71, 0.98)  *d* = 0.01 | 0.28 (−0.49, 1.05)  *d* = 0.02 | 0.14 (−0.70, 0.99)  *d* = 0.01 | 0.27 (−0.50, 1.05)  *d* = 0.02 |
| 2019 | −0.05 (−0.81, 0.71)  *d* = −0.00 | −0.12 (−0.80, 0.56)  *d* = −0.01 | −0.04 (−0.80, 0.72)  *d* = −0.00 | −0.12 (−0.80, 0.56)  *d* = −0.01 | −0.05 (−0.81, 0.99)  *d* = −0.00 | −0.13 (−0.81, 0.55)  *d* = −0.01 |
| 2020 | NA | NA | NA | NA | NA | NA |
| Constant | NA | NA | NA | NA | NA | NA |

^*^p < 0.05, ^**^p < 0.01.

^c^ Reference category: high school graduate or lower

^d^ Reference category: have not taken leave

^e^ Reference category: full-time

^f^ Reference category: manual

^g^ Reference category: 2007

**Table S4**

Between-person effect estimates of the covariates.

|  | Estimates (95% confidence interval)  Cohen’s *d* | | | | | |
| --- | --- | --- | --- | --- | --- | --- |
|  | Model 1 | | Model 2 | | Model 3 | |
|  | Men | Women | Men | Women | Men | Women |
| Age | −0.05 (−0.75, 0.65)  *d* = −0.00 | 0.30 (−0.33, 0.93)  *d* = 0.02 | −0.29 (−1.00, 0.41)  *d* = −0.02 | −0.01 (−0.66, 0.63)  *d* = −0.00 | −0.27 (−0.98, 0.44)  *d* = −0.02 | −0.09 (−0.74, 0.56)  *d* = −0.01 |
| Age squared | 0.00 (−0.01, 0.01)  *d* = 0.00 | −0.00 (−0.01, 0.01)  *d* = −0.00 | 0.00 (−0.01, 0.01)  *d* = 0.00 | 0.00 (−0.01, 0.01)  *d* = 0.00 | 0.00 (−0.01, 0.01)  *d* = 0.00 | 0.00 (−0.01, 0.01)  *d* = 0.00 |
| Educational attainment^c^ |  |  |  |  |  |  |
| Junior or community college graduates | −0.81 (−2.16, 0.54)  *d* = −0.05 | 1.30 (0.13, 2.47)^*^  *d* = 0.08 | −0.77 (−2.12, 0.58)  *d* = −0.05 | 1.40 (0.23, 2.57)^**^  *d* = 0.08 | −0.74 (−2.10, 0.61)  *d* = −0.04 | 1.40 (0.22, 2.57)^**^  *d* = 0.08 |
| College graduates or higher | 0.27 (−1.01, 1.55)  *d* = 0.02 | 1.82 (0.55, 3.09)^*^  *d* = 0.11 | 0.40 (−0.88, 1.68)  *d* = 0.02 | 2.09 (0.81, 3.37)^**^  *d* = 0.12 | 0.41 (−0.87, 1.69)  *d* = 0.02 | 2.14 (0.85, 3.42)^**^  *d* = 0.13 |
| Living standard | 4.22 (3.52, 4.92)^**^  *d* = 0.25 | 4.80 (4.13, 5.48)^**^  *d* = 0.29 | 4.05 (3.34, 4.75)^**^  *d* = 0.24 | 4.68 (4.00, 5.36)^**^  *d* = 0.28 | 4.02 (3.31, 4.72)^**^  *d* = 0.24 | 4.66 (3.98, 5.35)^**^  *d* = 0.28 |
| Maternity and childcare leave^d^ | −8.05 (−18.92, 2.82)  *d* = −0.48 | 5.39 (2.31, 8.47)^**^  *d* = 0.32 | −10.1 (−20.9, −0.81)^**^  *d* = −0.60 | 2.60 (−0.70, 5.91)  *d* = 0.15 | −10.4 (−21.4, −0.50)^**^  *d* = −0.62 | 2.47 (−0.87, 5.82)  *d* = 0.15 |
| Employment status^e^ |  |  |  |  |  |  |
| Part-time | −1.47 (−3.01, −0.08)  *d* = −0.09 | 0.10 (−1.05, 1.24)  *d* = 0.01 | −0.53 (−2.13, 1.06)  *d* = −0.03 | −0.44 (−1.61, 0.72)  *d* = −0.03 | −0.63 (−2.24, 0.99)  *d* = −0.04 | −0.55 (−1.72, 0.62)  *d* = −0.03 |
| Self-employed | −0.07 (−2.14, 2.01)  *d* = −0.00 | −1.55 (−4.13, 1.03)  *d* = −0.09 | 0.16 (−1.91, 2.23)  *d* = 0.01 | −2.02 (−4.60, 0.56)  *d* = −0.12 | 0.20 (−1.87, 2.27)  *d* = 0.01 | −2.09 (−4.68, −0.51)^**^  *d* = −0.12 |
| Occupation^f^ |  |  |  |  |  |  |
| Professional-managerial | −0.46 (−1.96, 1.04)  *d* = −0.03 | −1.97 (−3.80, −0.14)^*^  *d* = −0.12 | −0.66 (−2.16, 0.84)  *d* = −0.04 | −2.16 (−3.99, −0.32)^**^  *d* = −0.13 | −0.65 (−2.15, 0.86)  *d* = −0.04 | −2.16 (−4.00, −0.32)^**^  *d* = −0.13 |
| Non-manual | −1.89 (−3.15, −0.64)^**^  *d* = −0.11 | −1.35 (−2.96, 0.26)  *d* = −0.08 | −1.88 (−3.13, −0.63)^**^  *d* = −0.11 | −1.31 (−2.92, 0.29)  *d* = −0.08 | −1.86 (−3.12, −0.61)^**^  *d* = −0.11 | −1.36 (−2.97, 0.25)  *d* = −0.08 |
| Year dummy^g^ |  |  |  |  |  |  |
| 2008 | −0.76 (−5.16, 3.63)  *d* = −0.05 | −1.58 (−6.66, 3.50)  *d* = −0.09 | −0.40 (−4.79, 3.98)  *d* = −0.02 | −1.17 (−6.25, 3.90)  *d* = −0.07 | −0.40 (−4.80, 3.99)  *d* = −0.02 | −1.24 (−6.34, 3.85)  *d* = −0.07 |
| 2009 | −6.12 (−11.3, −0.93)^*^  *d* = −0.36 | −0.23 (−6.61, 6.14)  *d* = −0.01 | −5.85 (−11.0, −0.67)^**^  *d* = −0.35 | −0.17 (−6.53, 6.20)  *d* = −0.01 | −5.77 (−11.0, −0.59)^**^  *d* = −0.34 | 0.12 (−6.26, 6.51)  *d* = 0.01 |
| 2010 | −3.19 (−10.5, 4.08)  *d* = −0.19 | −6.37 (−13.2, −0.43)  *d* = −0.38 | −3.65 (−10.9, 3.59)  *d* = −0.22 | −6.37 (−13.2, 0.42)  *d* = −0.38 | −3.64 (−10.9, 3.62)  *d* = −0.22 | −6.32 (−13.1, −0.48)^**^  *d* = −0.38 |
| 2011 | −1.50 (−5.28, 2.27)  *d* = −0.09 | 4.49 (0.19, 8.80)^*^  *d* = 0.27 | −1.48 (−5.25, 2.28)  *d* = −0.09 | 4.83 (0.53, 9.13)^**^  *d* = 0.29 | −1.74 (−5.52, 2.05)  *d* = −0.10 | 4.87 (0.55, 9.18)^**^  *d* = 0.29 |
| 2012 | 1.77 (−5.85, 9.39)  *d* = 0.10 | −0.17 (−6.98, 6.63)  *d* = −0.01 | 1.73 (−5.86, 9.33)  *d* = 0.10 | −0.06 (−6.85, 6.73)  *d* = −0.00 | 1.73 (−5.88, 9.35)  *d* = 0.10 | 0.37 (−6.45, 7.18)  *d* = 0.02 |
| 2013 | −2.64 (−11.5, 6.18)  *d* = −0.16 | −6.21 (−14.2, 1.80)  *d* = −0.37 | −2.13 (−10.9, 6.65)  *d* = −0.13 | −6.02 (−14.0, 1.98)  *d* = −0.36 | −2.09 (−10.9, 6.73)  *d* = −0.12 | −5.96 (−14.0, 2.06)  *d* = −0.36 |
| 2014 | 8.29 (−2.31, 18.9)  *d* = 0.49 | −0.95 (−9.25, 7.35)  *d* = −0.06 | 8.63 (−1.94, 19.20)  *d* = 0.51 | −1.00 (−9.28, 7.28)  *d* = −0.06 | 8.71 (−1.88, 19.3)  *d* = 0.52 | −0.68 (−8.99, 7.28)  *d* = −0.04 |
| 2015 | −11.4 (−22.2, −0.68)^*^  *d* = −0.68 | 6.93 (−0.79, 14.7)  *d* = 0.41 | −10.8 (−21.5, −0.04)^**^  *d* = −0.64 | 7.12 (−0.60, 14.8)  *d* = 0.42 | −11.0 (−21.7, −0.21)^**^  *d* = −0.65 | 6.90 (−0.83, 14.6)  *d* = 0.41 |
| 2016 | −10.7 (−23.5, −2.07)  *d* = −0.64 | 6.21 (−4.50, 16.9)  *d* = 0.37 | −9.55 (−22.3, −3.22)^**^  *d* = −0.57 | 5.70 (−5.00, 16.4)  *d* = 0.34 | −9.79 (−22.6, −3.01)^**^  *d* = −0.58 | 5.57 (−5.16, 16.3)  *d* = 0.33 |
| 2017 | 19.7 (3.67, 35.8)^*^  *d* = 1.17 | −1.07 (−12.9, 10.7)  *d* = −0.06 | 17.7 (1.72, 33.8)^**^  *d* = 1.05 | −0.34 (−12.1, 11.4)  *d* = −0.02 | 18.2 (2.06, 34.3)^**^  *d* = 1.08 | −0.26 (−12.1, 11.6)  *d* = −0.02 |
| 2018 | −9.28 (−22.2, 3.60)  *d* = −0.55 | 2.30 (−5.56, 10.2)  *d* = 0.14 | −8.44 (−21.3, −4.40)^**^  *d* = −0.50 | 1.14 (−6.73, 9.00)  *d* = 0.07 | −8.61 (−21.5, −4.26)^**^  *d* = −0.51 | 1.08 (−6.81, 8.97)  *d* = 0.06 |
| 2019 | −1.72 (−4.24, 0.81)  *d* = −0.10 | −0.96 (−3.67, 1.74)  *d* = −0.06 | −1.68 (−4.20, 0.84)  *d* = −0.10 | −0.76 (−3.46, 1.95)  *d* = −0.04 | −1.69 (−4.21, 0.84)  *d* = −0.10 | −0.64 (−3.36, 2.07)  *d* = −0.04 |
| 2020 | −1.92 (−4.89, 1.04)  *d* = −0.11 | −0.22 (−3.16, 2.71)  *d* = −0.01 | −1.64 (−4.60, 1.32)  *d* = −0.10 | −0.16 (−3.09, 2.77)  *d* = −0.01 | −1.58 (−4.55, 1.39)  *d* = −0.09 | −0.19 (−3.13, 2.75)  *d* = −0.01 |
| Constant | 49.7 (36.9, 62.4)^**^  *d* = 2.94 | 37.1 (25.5, 48.6)^**^  *d* = 2.23 | 53.9 (41.2, 66.7)^**^  *d* = 3.19 | 43.2 (31.4, 55.0)^**^  *d* = 2.57 | 53.8 (41.0, 66.7)^**^  *d* = 3.18 | 44.8 (32.9, 56.8)^**^  *d* = 2.68 |

^*^p < 0.05, ^**^p < 0.01.

^c^ Reference category: high school graduate or lower

^d^ Reference category: have not taken leave

^e^ Reference category: full-time

^f^ Reference category: manual

^g^ Reference category: 2007
